# Genome-wide Association Studies for Female Fertility Traits in Chinese and Nordic Holsteins

**DOI:** 10.1038/s41598-017-09170-9

**Published:** 2017-08-16

**Authors:** Aoxing Liu, Yachun Wang, Goutam Sahana, Qin Zhang, Lin Liu, Mogens Sandø Lund, Guosheng Su

**Affiliations:** 1 Laboratory of Animal Genetics, Breeding and Reproduction, Ministry of Agriculture of China, National Engineering Laboratory of Animal Breeding, College of Animal Science and Technology, China Agricultural University, Beijing, 100193 China; 2 Center for Quantitative Genetics and Genomics, Department of Molecular Biology and Genetics, Aarhus University, 8830 Tjele, Denmark; 3 Beijing Dairy Cattle Center, Beijing, 100192 China

## Abstract

Reduced female fertility could cause considerable economic loss and has become a worldwide problem in the modern dairy industry. The objective of this study was to detect quantitative trait loci (QTL) for female fertility traits in Chinese and Nordic Holsteins using various strategies. First, single-trait association analyses were performed for female fertility traits in Chinese and Nordic Holsteins. Second, the SNPs with *P*-value < 0.005 discovered in Chinese Holsteins were validated in Nordic Holsteins. Third, the summary statistics from single-trait association analyses were combined into meta-analyses to: (1) identify common QTL for multiple fertility traits within each Holstein population; (2) detect SNPs which were associated with a female fertility trait across two Holstein populations. A large numbers of QTL were discovered or confirmed for female fertility traits. The QTL segregating at 31.4~34.1 Mb on BTA13, 48.3~51.9 Mb on BTA23 and 34.0~37.6 Mb on BTA28 shared between Chinese and Nordic Holsteins were further ascertained using a validation approach and meta-analyses. Furthermore, multiple novel variants identified in Chinese Holsteins were validated with Nordic data as well as meta-analyses. The genes *IL6R*, *SLC39A12*, *CACNB2*, *ZEB1*, *ZMIZ1* and *FAM213A* were concluded to be strong candidate genes for female fertility in Holsteins.

## Introduction

Poor female fertility has become a serious worldwide problem in modern dairy cattle industry, due to its unfavorable genetic correlations with milk yield traits which have gone through intensive selections during the past few decades^1^. Poor female fertility could cause considerable economic loss as a consequence of increased additional inseminations, veterinary treatments and involuntary culling. Nordic countries are well known for putting higher economic weights on female fertility traits in the selection index, and are also among the countries of highest genetic levels on fertility^2^. A recent investigation, however, suggested that genetic progresses for female fertility traits in Nordic countries were still unsatisfactory^3^, which might be because the traits have low heritability, unfavorable genetic correlations with milk yield traits and are measured late in life^4^.

Faster genetic progress for female fertility could be achieved by incorporating the information of QTL into selection decisions. With the help of high-density single nucleotide polymorphism (SNP) panel, genome-wide association studies (GWAS) have been used as primary strategies to detect QTL for complex traits since 2005^5^. Hundreds of genetic variants were reported to be associated with female fertility in various Holstein populations: US Holsteins (1,654 cows)^6^, Danish and Sweden Holsteins (2,531 bulls)^7^, Germany Holsteins (2,527 bulls)^8^, and Italian Holsteins (2,139 bulls)^9^. In Nordic Holsteins, a series of QTL mapping studies on female fertility have been previously carried out^4, 7, 10, 11^. For example, 24 significant QTL were detected on 14 different chromosomes for 11 fertility-related traits using Illumina BovineSNP50 BeadChip (54 K) SNP chip in Danish and Swedish Holsteins^7^ and a sharp peak was observed on BTA13 for interval from calving to first insemination. The QTL on BTA4 and BTA13 were further narrowed down by fine mapping using the 54k genotypes imputed to the high density SNP array (Illumina BovineHD BeadChip, 777 K) and further to whole genome sequence variants^10, 11^. Nevertheless, the results obtained from various association studies did not overlap very well across populations. This could be explained by the inconsistent trait definitions among different countries, possible differences in linkage disequilibrium (LD) structures across populations, and insufficient QTL detection power due to low heritabilities and small sample sizes^10^.

In Chinese Holsteins, reference animals with both phenotype and genotype are limited and primarily consist of cows. Compared with proven bulls, cows usually have lower reliabilities for pseudo phenotypes (such as daughter yield deviations (DYD)). Moreover, a stringent threshold, such as Bonferroni correction for multiple tests, was widely used in GWAS to control the false positives. Therefore, it is difficult to discover the genetic variants underling female fertility in Chinese Holsteins directly. A promising solution is to use information from other populations, especially genetically closely related populations^10^. One strategy is to use a suggestive threshold of significance in an association study of one population and then validate candidate SNPs in other populations, since the probability of a SNP being significant by chance in two populations is low^12^. For example, the QTL on female fertility discovered in Nordic Holsteins were validated in Nordic Red and Jersey^10^. An alternative approach is to use meta-analysis, which can increase the power by pooling the summary statistics of multiple independent studies together^13^. Compared with the conventional multi-trait model, raw phenotype and genotype data at individual level are not required for meta-analysis. Thus, the barrier of sharing raw data among different research institutes is removed, and inconsistence of trait definitions and sample sizes are also allowed for. The meta-analysis can be implemented either for the same trait across populations or for multiple traits within a population. Previous studies performed in dairy cattle on growth^14^ and longevity^15^ traits have already shown that novel QTL were detected using meta-analysis across populations. In addition, Bolormaa *et al*.^16^ showed that the detection power, which was measured by numbers of SNPs validated in an independent population, for stature, fatness and reproduction in beef cattle was increased using meta-analysis for multiple traits within population.

The relationship between Chinese and Nordic Holstein populations have been widely investigated in previous studies^17–19^. The LD phase between Chinese and Nordic Holsteins was in high consistency, and reliabilities of genomic prediction for production traits in Chinese Holsteins increased using the joint Chinese-Nordic reference population^17^. With the help of Nordic Holsteins, the accuracy of imputation from 54 K to 777 K was also increased in Chinese Holsteins^18^. In 2015, a joint GWAS for milk fatty acid traits was performed using Illumina BovineHD BeadChip markers from 784 Chinese and 371 Danish Holstein cows, a large number of additional significant SNPs were detected^19^. Therefore, we hypothesized that meta-analysis within or across populations and validation using information of Nordic Holsteins could be helpful for QTL detection for female fertility traits in Chinese Holsteins.

There were two main objectives in the present study. The first was to detect QTL for female fertility traits in Chinese and Nordic Holsteins using various strategies and methods, including conventional single-trait GWAS within population, validation of SNPs detected in Chinese Holsteins using Nordic Holstein data, and meta-analyses for multiple traits within a population and for the same trait within or across populations. The second was to test the hypothesis of increasing power by meta-analysis and validation using information from other population.

## Materials and Methods

### Ethics statement

All phenotypic data were recorded as part of routine dairy cattle management and genetic evaluations. The DNA samples were obtained for the purpose of routine genomic evaluations in previous projects. Thus, no additional animal handling or experiment was performed specifically for this study.

### Animals and traits

Data of female fertility traits from Chinese and Nordic Holsteins were analyzed in this study. There were a total of 4,555 genotyped Chinese Holsteins, including 167 bulls and 4,388 cows. The following traits were analyzed: age at first insemination **(AFS)**, interval from calving to first insemination (**ICF**), days open (**DO**), interval from first to last insemination (**IFL**), conception rate at first insemination (**CR**), number of inseminations per conception (**AIS**), non-return rate within 56 d after first insemination (**NRR56**). For binary traits, CR was coded as 0 (failure) or 1 (success), and NRR56 was coded as 1 when there was no subsequent insemination within 56 d after the first insemination and 0 otherwise. The performances of heifers and cows (lactation 1 to 3) were considered as different traits, thus, h and c were used as suffixes in trait abbreviations accordingly. Among these traits, AFS was available only for heifers; ICF and DO were available only for cows. All the remaining traits were measured for both heifers and cows.

Data of 7,048 bulls from Denmark, Sweden and Finland were used for detecting QTL on female fertility in Nordic Holsteins. Four traits, CRh, ICF, IFLc and CRc, which were used for both Nordic routine evaluation and Multiple Across Country Evaluation (**MACE**) by Interbull (http://www.interbull.org/index), were analyzed in this study. The trait definitions of CRh, ICF, IFLc and CRc in Nordic Holsteins were similar to those implemented in Chinese Holsteins. Additional details regarding trait definitions in Nordic Holsteins can be found at the official website (http://www.nordicebv.info/).

### Phenotype information for GWAS

In Chinese Holsteins, DYD of bulls and yield deviation (YD) of cows for 11 female fertility traits were used as “pseudo” phenotypes for GWAS. The DYD and YD were calculated using a single-trait animal model^20^ based on the full dataset of the population. The full data included 215,632 field records on 88,647 females over the period from 2000 to 2014 and pedigree data of 184,249 animals. Cow traits were analyzed by pooling data over parities and treating records in multiple parities as repeated measurements. Fixed effects were age group (in terms of AFS, divided as 270~439 d, 440~469 d, 470~499 d, 500~529 d and 530~900 d) for all traits excluding AFS itself, herd-year of birth (for AFS), herd- year of calving (for ICF and DO) or herd-year of first insemination within parity (for IFL, CR, AIS and NRR56 in both heifers and cows), year-month of birth (for AFS), year-month of calving (for ICF and DO) or year-month of first insemination within parity (for IFL, CR, AIS and NRR56 in both heifers and cows), AI technician (for all traits), use of sexed semen (for all traits except AFS, ICF and DO), parity (for all cow traits). Besides, random permanent environment effects were also included in the model for all cows traits. Estimates of variance components and heritabilities for female fertility traits in Chinese Holsteins are shown in Supplementary Table S1. To avoid the double counting in GWAS, the phenotypic values of genotyped daughters was excluded when estimating the DYD of genotyped bulls. The above models were accomplished applying the average information-restricted maximum likelihood (AI-REML) implemented in the DMU package^21^.

The de-regressed proof (DRP) of female fertility traits were used for association study in Nordic Holsteins. The DRP of Nordic Holsteins were derived from Nordic genetic evaluations. Further details regarding the data editing, models and genetic parameters could be found in the previous studies for Chinese Holsteins^22^ and Nordic Holsteins (http://www.nordicebv.info).

### Genotypes

Chinese and Nordic Holsteins were genotyped using Illumina BovineSNP50 BeadChip (Illumina Inc., San Diego, CA, USA) version 1 (containing 54,001 SNPs) or version 2 (containing 54,609 SNPs). There are 52,340 common SNPs in both version and 56,270 SNPs in total. The same SNP quality control criteria were used for both Holstein populations, i.e.: SNPs were removed if the minor allele frequency (MAF) was less than 1%, or a significant deviation from the Hardy-Weinberg equilibrium with a *P*-value below 10^−5^. The SNPs with unknown position and on the X chromosome were excluded from this study. To construct the same set of SNPs, genotype imputation was performed in Chinese Holsteins by BEAGLE software^23^. For Nordic bulls, no imputation was performed in the genotype data, thus, only animals with call rate higher than 90% were kept. After editing, 44,462 SNPs of 4,555 individuals were available for association tests in Chinese Holsteins, while 44,692 SNPs of 7,084 bulls were available in Nordic Holsteins, and 42,933 SNPs were common in the two populations. The UMD version 3.1 assembly^24^ was used as the reference for the SNP position.

### Single-trait GWAS

The association analysis was performed using a linear mixed model analysis (MMA)^25^ implemented in the DMU package^21^. The SNPs were fitted to the model one by one:

1 $${\boldsymbol{y}}={\bf{1}}\mu +{\rm{b}}{\boldsymbol{x}}+{\boldsymbol{Za}}+{\boldsymbol{e}}$$

where ${\boldsymbol{y}}$ was the vector of pseudo phenotypes for female fertility traits, $1$ was the vector of ones, ${\boldsymbol{\mu }}$ was the general mean, ${\rm{b}}$ was the additive allele substitution effect of the SNP, ${\boldsymbol{x}}$ was the vector of allele dosages (coded as 0, 1 or 2) for one randomly chosen allele of the SNP, ${\boldsymbol{Z}}$ was the incidence matrix connecting additive polygenic effects to corresponding pseudo phenotype, ${\boldsymbol{a}}$ was the vector of additive polygenic effects and ${\boldsymbol{e}}$ was the vector of random residuals. It was assumed that ${\boldsymbol{a}}$ follows a normal distribution $N(0,\,{\boldsymbol{A}}{\sigma }_{a}^{2})$, in which ${\boldsymbol{A}}$ was the matrix of additive genetic relationships between individuals built with pedigree information and ${\sigma }_{a}^{2}$ was the polygenic variance. For random residuals, it was assumed that ${\boldsymbol{e}} \sim N(0,\,{{\boldsymbol{W}}}^{-1}{\sigma }_{e}^{2})$, where ${\sigma }_{e}^{2}$ was the residual variance and ${\boldsymbol{W}}$ was the diagonal matrix containing the element of ${w}_{i}$ which was the weight on the $i\,$th pseudo phenotype. The weight for the pseudo phenotype (DYD/YD for Chinese Holsteins or DRP for Nordic Holsteins) of the $i\,$th animal was calculated as ${w}_{i}=\,{r}_{i}^{2}/(1-{r}_{i}^{2})$, in which, ${r}_{i}^{2}$ was the reliability of the $i\,$th pseudo phenotype, and ${r}_{i}^{2}$ higher than 0.98 was set to 0.98 to avoid extremely large weight on the pseudo phenotype for individuals with very high reliabilities. The variance components ${\sigma }_{a}^{2}$ and ${\sigma }_{e}^{2}\,$were estimated in each run. The sampling variance for allele substitution effect $Var(\hat{{\boldsymbol{b}}})$ was obtained via the mixed model equations. A Wald chi-squared statistic ${b}^{2}/Var(\hat{b})$ with ${\rm{df}}=1$ was constructed to examine whether the SNP was associated with the trait.

A Bonferroni correction was used for correcting the multiple testing in the within-population single-trait GWAS. Significance thresholds corresponding to a nominal 5% type I error was applied in this study, which was defined as 0.05/N, where N was the number of SNP loci. As the number of SNPs in two Holstein populations were rather similar, the same genome-wide significance threshold of $1.12\times {10}^{-6}$ was applied for single-trait GWAS in both populations. Moreover, a less stringent chromosome-wide significance was also used for Chinese Holsteins, with suggestive significance thresholds from $1.73\times {10}^{-5}$ on BTA1 to $5.94\times {10}^{-5}$ on BTA27.

### Validate candidate SNPs from Chinese Holsteins using Nordic Holstein data

Due to low heritabilities of female fertility traits, the low reliability of DYD for cows, and the stringent Bonferroni correction, a relatively low power of detecting QTL in the Chinese Holstein population is expected. Thus, an alternative approach was applied to increase both the power and the precision of QTL detection in Chinese Holsteins by validating SNP of interest in Nordic Holsteins. In this approach, the SNP with *P*-value < 0.005 discovered in Chinese Holsteins were preliminarily selected as candidates and further validated by association analysis in Nordic Holsteins for the corresponding trait and applying Bonferroni correction for the number of SNPs validated.

### Multi-trait meta-analysis within population

Three multi-trait meta-analyses were carried out to discover common QTL for traits manifesting similar fertility function and to further increase the power of detecting QTL within population. In general, pleiotropy is usually used to indicate a gene or a specific allele which influences more than one phenotype, especially for seemingly unrelated phenotypic traits^26^. In the present study, the traits reflecting similar fertility function can be considered as different traits with some common genetic components. Thus in the context, we specifically defined the QTL with common effect on different measures of similar fertility ability as common QTL. The performances of a trait in heifers and cows were typically considered as different due to low genetic correlations^27^. Therefore, the meta-analysis was only performed within heifer traits or within cow traits instead pooling all fertility traits together. In Chinese Holsteins, the first meta-analysis was performed for IFLh, CRh, AISh and NRR56h, which measured the abilities of the maiden heifers to conceive and maintain pregnancy. AFS was excluded from the analysis of heifers since the effects of AFS have already been utilized when estimating DYD for other fertility traits. The second meta-analysis was performed for ICF, DO, IFLc, CRc, AISc and NRR56c, which measured the abilities of lactating cows to recycle after calving or capacities to conceive and maintain pregnancy. Within Nordic Holsteins, a meta-analysis was performed only for three cow traits, which were ICF, IFLc and CRc.

An approximated chi-square statistic^16^ was applied to test whether there is at least one of the SNP effect of studied traits was not equal to zero. For each SNP, chi-square statistic of a multi-trait meta-analysis was calculated using the following formula:

2 $${\chi }^{2}={t}_{i}^{\text{'}}{V}^{-1}{t}_{i}$$

where ${t}_{i}$ was a vector of signed *t-*values of the $i\,$th SNP for all studied traits, ${t}_{i}^{\text{'}}$ was a transpose of the vector ${t}_{i}$, ${V}^{-1}$ was an inverse of the correlation matrix where the correlation between a pair of traits was estimated from the correlation of summary statistics (signed *t*-value from single-trait GWAS) over the SNPs in the analysis. To avoid inflated correlation estimates caused by relationships among SNPs, independent SNPs across the whole genome were selected on the basis of LD pattern to calculate the correlations between traits^28^. In this study, independent SNPs were chosen using the following approach^29^. Every contiguous 100 SNPs within the same chromosome were considered as a window, and no overlap was shared by two adjacent windows. The threshold of variance inflation factor (VIF) was set to 2 and was calculated as: ${\rm{VIF}}=1/(1-{r}^{2})$, where ${r}^{2}$ was the square of the coefficient of multiple correlation (i.e., the coefficient of determination) for a SNP being regressed on all other SNPs in the window simultaneously. The SNP with VIF larger than 2 was excluded from the calculation of correlations^29^. The tag SNP pruning was carried out using the software Plink^30^. The significance threshold from single-trait GWAS was applied for the *P*-value of multi-trait meta-analysis within population.

### Meta-analysis across populations

Meta-analyses across populations was performed for the traits (CRh, ICF, CRc and IFLc) which were analyzed using single-trait in both Chinese and Nordic Holsteins, separately. Association evidences from two individual studies were pooled together with appropriate weights. The sample size weighted Z-score method was conducted in this study using software METAL^31^, which combines the *P*-value and the direction of SNP effect obtained in each independent study. First, the overall Z-score was calculated as:

3 $$Z=\frac{{\sum }_{i}{Z}_{i}{W}_{i}}{\sqrt{{\sum }_{i}{w}_{i}^{2}}},$$

where ${Z}_{i}={\Phi }^{-1}(\frac{{P}_{i}}{2})\ast {\rm{sign}}({{\rm{\Delta }}}_{i})$; ${W}_{i}=\sqrt{{N}_{i}}$; $i$ was the $i\,$th study; ${P}_{i}$ was the *P*-value of SNP; $\Phi$ was the cumulative distribution function of the standard normal distribution; ${{\rm{\Delta }}}_{i}$ was the direction of SNP effect; ${N}_{i}$ was the sample size. Ultimately, the overall *P*-value was calculated as $P=2\Phi (-|Z|)$. The pre-correction to *P*-values using genomic control^32^ was applied for meta-analysis across populations.

### The QTL region and the explained variance

#### The definition of QTL region

A QTL region was subjectively defined by extending from the position of the most significant SNP (top SNP) within a peak to the both side until all extended SNPs within that region had a $-{\mathrm{log}}_{10}$(*P*-value) higher than $-{\mathrm{log}}_{10}$(*P*-value) of the top SNP minus three^14^.

#### The variance explained by QTL

The proportion of variance explained by the most significant SNP within a QTL was calculated as $2pq{b}^{2}/{\sigma }_{a}^{2}$, where $p$ and $q$ were allele frequencies, $b$ was the allele substitution effect, and ${\sigma }_{a}^{2}$ was the genetic variance of the trait^33^.

### Test for multiple QTL

From the results of single-trait GWAS, multiple SNPs were detected within the same chromosome for the same fertility trait in both Chinese and Nordic Holsteins. To test whether there were multiple QTL segregating within a chromosome, the most significant SNP within the chromosome observed from single-trait GWAS was fitted into MMA as a cofactor. Each of the remaining SNPs within the same chromosome was successively examined, respectively.

4 $${\boldsymbol{y}}=1{\boldsymbol{\mu }}+{b}_{top}{\boldsymbol{SN}}{{\boldsymbol{P}}}^{{\boldsymbol{top}}}+b{\boldsymbol{x}}+{\boldsymbol{Za}}+{\boldsymbol{e}}$$

where ${b}_{top}$ was the additive allele substitution effect of the most significant SNP, and ${\boldsymbol{SN}}{{\boldsymbol{P}}}^{{\boldsymbol{top}}}$ was the vector of allele dosages for the most significant SNP. Other components in model (4) were the same as in model (1). The significant threshold was the same as that used for single-trait GWAS.

### Data availability

Data supporting this paper were obtained from the commercial dairy farms in China and Nordic countries. The phenotype and genotype data are available only upon agreement with commercial breeding organizations and should be requested directly from the corresponding authors or the breeding organizations.

## Results

### Reliabilities of pseudo phenotypes

The numbers of animals and the corresponding reliabilities of pseudo phenotype for each trait in each population are listed in Table 1. The average reliabilities for DYD of Chinese bulls ranged from 0.348 for NRR56h to 0.889 for AFS, while the average reliabilities for YD of Chinese cows were much lower, from 0.008 for NRR56h to 0.447 for AFS. Meanwhile, the average reliabilities for DRP of Nordic bulls were in a range of 0.612 for CRh to 0.702 for CRc.

**Table 1:** Number of animals and average reliabilities of pseudo phenotypes for female fertility trait in Chinese and Nordic Holsteins.

**Trait** ^1^	**Chinese Holsteins**				**Nordic Holsteins**	
	N^2^(cow)	Reliability^3^(cow)	N^2^(bull)	Reliability^3^(bull)	N(bull)^2^	Reliability^3^
**AFS**	4,368	0.447	167	0.889	—	—
**IFLh**	4,368	0.011	167	0.382	—	—
**CRh**	4,368	0.010	167	0.372	5,909	0.612
**AISh**	4,368	0.012	167	0.391	—	—
**NRR56h**	4,368	0.008	167	0.348	—	—
**ICF**	4,140	0.175	165	0.722	6,178	0.690
**DO**	4,140	0.098	165	0. 627	—	—
**IFLc**	4,140	0.065	165	0.567	6,178	0.690
**CRc**	4,140	0.027	165	0.451	6,112	0.702
**AISc**	4,140	0.063	165	0.562	—	—
**NRR56c**	4,140	0.014	165	0.372	—	—

^1^AFS = age at first insemination; IFL = interval from first to last insemination; CR = conception rate at first insemination; AIS = number of inseminations per conception; NRR56 = non-return rate at 56 d after first insemination; ICF = interval from calving to first insemination; DO = days open. For the traits expressed in both heifers and cows, a suffixes h (for heifers) or c (for cows) was attached to the trait abbreviations.

^2^The number of animals.

^3^The average reliability of pseudo phenotypes (yield deviation for cows, daughter yield deviation for Chinese bulls and de-regressed proof for Nordic bulls).

### GWAS in Chinese Holsteins

Manhattan plots for female fertility traits in Chinese Holsteins are presented in Supplementary Fig. S1. There were three SNPs above the genome-wide significant level but with low MAF (below 0.05), including rs110304855 for NRR56h, rs517592568 and rs110777693 for AISh. Besides, 47 additional SNPs were chromosome-wide significantly associated with female fertility traits in Chinese Holsteins. The summary of QTL detected on chromosome-wide level for Chinese Holsteins is shown in Supplementary Table S2. A total of 34 QTL were identified across all traits, of which eight QTL were associated with more than one trait. Genes harboring or closest to the top SNPs were defined as candidate genes. In total, 51 protein-coding genes were identified as potential candidates for female fertility traits in Chinese Holsteins.

### GWAS in Nordic Holsteins

Manhattan plots for female fertility traits in Nordic Holsteins are presented in Supplementary Fig. S2. Compared with GWAS results for Chinese Holsteins, many more significant SNPs were found in Nordic Holsteins. In total, 334 SNPs were genome-wide significantly associated with female fertility traits, of which 91 SNPs were associated with more than one trait. The most significantly associated SNP was observed for ICF (rs41574065, *P*-value = $3.75\times {10}^{-17}$), which explained 1.14% of the total additive genetic variance. This SNP was also significant for IFLc. A summary of genome-wide significant QTL detected in Nordic Holstein is listed in Supplementary Table S3. A total of 48 QTL were defined in Nordic Holsteins, of which 24 QTL harbored more than one significant SNP. Among all QTL, eight QTL were found to be significantly associated with multiple fertility traits. In total, 72 candidate genes were identified, of which 71 genes were protein-coding genes.

### Validate candidate SNPs from Chinese Holsteins using Nordic Holstein data

The number of SNPs detected in Chinese Holsteins available for validation in Nordic Holsteins differed across traits. There were 335 (CRh), 409 (ICF), 506 (IFLc) and 299 (CRc) SNPs with *P*-value < 0.005 in Chinese Holsteins selected for validation using the Nordic Holstein data. The corresponding thresholds for Bonferroni correction for validation in Nordic Holsteins were $1.49\times {10}^{-4}$ (CRh), $1.22\,\times {10}^{-4}$(ICF), $9.88\,\times {10}^{-5}$ (IFLc) and $1.67\times {10}^{-4}$ (CRc), respectively. At the end, 37 SNPs were confirmed in Nordic Holsteins (see Table 2), of which 30 were not significant in previous single-trait GWAS for either of the populations. In total, 41 additional candidate genes were further identified using this validation approach.

**Table 2:** The SNPs associated with female fertility traits detected by validating candidate SNPs from Chinese Holsteins using Nordic Holstein data.

BTA	Position^1^(bp)	SNP	Trait^2^	MAF^3^	Chinese Holsteins		MAF^3^	*Nordic Holsteins*		Genes^5^
					*P*-value	Effect size^4^(%)		*P*-value	Effect size^4^(%)	
3	16,179,998	rs109155222	IFLc	0.40	4.33E-03	1.75	0.43	3.41E-05	0.30	*IL6R*
3	25,763,148	rs110094798	IFLc	0.24	3.30E-03	1.95	0.20	3.12E-05	0.35	*FAM46C, MAN1A2*
3	26,949,196	rs109368837	IFLc	0.22	2.78E-03	2.09	0.20	7.67E-05	0.31	*CD58, ATP1A1*
3	66,768,945	rs42361598	IFLc	0.42	3.94E-03	1.69	0.44	4.85E-05	0.31	*PTGFR, GIPC2*
4	47,737,653	rs110127380	CRh	0.47	4.40E-03	3.69	0.41	7.90E-05	0.37	*NAMPT, PIK3CG*
4	67,145,895	rs41589544	ICF	0.07	1.51E-04	0.85	0.12	5.19E-05	0.21	*CHN2*
6	87,600,892	rs109377247	IFLc	0.33	6.51E-04	2.31	0.33	8.67E-06	0.35	*ENSBTAG00000007816, AMTN*
10	29,538,245	rs42563243	ICF	0.44	2.62E-03	0.65	0.38	4.01E-05	0.32	*ENSBTAG00000021414 ENSBTAG00000025634*
10	83,435,322	rs43642952	CRc	0.16	3.07E-03	1.56	0.18	1.41E-05	0.29	*ENSBTAG00000005395, ENSBTAG00000025385*
11	83,938,054	rs29026760	ICF	0.26	3.70E-03	0.62	0.28	5.08E-05	0.28	*FAM84A, ENSBTAG00000019284*
11	85,906,433	rs110195627	ICF	0.23	2.90E-03	0.66	0.26	1.35E-05	0.31	*TRIB2, LPIN1*
13	30,713,329	rs109193608	IFLc	0.35	2.68E-03	1.89	0.42	7.55E-06	0.33	*FAM188A, PTER*
13	31,364,982	rs110068451	ICF, IFLc	0.34	3.44E-03, 9.21E-04	0.58, 2.18	0.41	6.70E-11, 1.33E-06	0.69, 1.12	*RSU1*
13	31,416,451	rs109492780	IFLc	0.38	5.90E-04	2.53	0.43	8.11E-09	0.56	*RSU1*
13	31,524,626	rs41604666	IFLc	0.49	4.41E-04	2.64	0.48	4.37E-07	0.46	*RSU1*
13	32,802,426	rs41609782	IFLc	0.40	3.26E-03	1.82	0.41	1.69E-05	0.31	*SLC39 A12, CACNB2*
13	32,831,454	rs109983109	ICF, IFLc	0.41	8.03E-04, 3.11E-03	0.81, 1.86	0.45	6.64E-10, 4.95E-06	0.66, 0.34	*SLC39A12, CACNB2*
13	33,501,056	rs109621404	IFLc	0.46	3.90E-03	1.95	0.40	1.65E-08	0.60	*EPC1*
13	52,447,531	rs109573317	CRc	0.35	4.58E-03	1.37	0.30	4.07E-05	0.29	*SLC4A11*
15	27,235,378	rs110869861	ICF	0.27	4.94E-03	0.50	0.23	9.02E-06	0.42	*CADM1, BUD13*
16	53,070,045	rs41580862	ICF	0.27	4.14E-04	0.84	0.26	8.50E-05	0.30	*SLC25A34*
16	53,097,936	rs110199510	ICF	0.27	5.89E-04	0.79	0.26	8.50E-05	0.30	*PLEKHM2*
16	57,419,757	rs41581444	ICF	0.31	5.01E-04	0.87	0.36	3.89E-05	0.30	*ENSBTAG00000027809, GPR52*
17	67,245,920	rs41848120	CRh	0.48	1.28E-04	7.32	0.46	3.87E-05	0.41	*PIWIL3*
17	72,448,619	rs41634418	ICF	0.35	3.57E-03	0.59	0.34	5.79E-06	0.40	*PISD*
18	48,150,900	rs110543856	ICF, IFLc	0.15	2.60E-03, 2.99E-04	0.65, 2.80	0.13	5.84E-08, 3.96E-07	0.61, 0.50	*SIPA1L3*
21	41,383,298	rs29012716	ICF	0.16	4.96E-04	0.81	0.12	5.23E-05	0.40	*PRKD1, G2E3*
21	47,273,394	rs110964123	ICF	0.40	2.13E-03	0.63	0.38	7.52E-05	0.30	*NKX2-8, PAX9*
23	50,036,208	rs110076231	CRc	0.38	1.27E-03	1.95	0.45	1.31E-04	0.25	*PRPF4B, PXDC1*
23	51,938,161	rs29027634	IFLc, CRc	0.36	3.25E-03, 4.73E-03	1.84, 1.45	0.37	8.89E-05,1.26E-04	0.27, 0.26	*EXOC2*
24	30,468,614	rs109722348	ICF	0.39	2.98E-03	0.66	0.38	6.27E-05	0.31	*AQP4, KCTD1*
24	30,513,490	rs110839120	ICF	0.39	2.65E-03	0.68	0.38	1.07E-04	0.28	*AQP4, KCTD1*
24	33,624,891	rs109629413	ICF	0.48	2.35E-03	0.62	0.47	7.81E-05	0.30	*TMEM241*

^1^The base pair position in the BTA (*Bos taurus* autosome).

^2^CRh = conception rate at first insemination in heifers; ICF = interval from calving to first insemination; IFLc = interval from first to last insemination in cows; CRc = conception rate at first insemination in cows.

^3^Minor allele frequency of the validated SNP.

^4^The percentage of genetic variance explained by the most significant SNP.

^5^Genes harbor or closest to the significant SNP.

### Multi-trait meta-analysis within population

A total of 10,749 and 10,546 SNPs were used as tag SNPs to construct the genomic correlation matrices between fertility traits in Chinese and Nordic Holsteins, separately. The absolute values of genomic correlations ranged from 0.29 to 0.81 for heifer traits in Chinese Holsteins, from 0.04 to 0.95 for cow traits in Chinese Holsteins, and from 0.33 to 0.90 for cow traits in Nordic Holsteins. Manhattan plots for meta-analysis within population are presented in Supplementary Fig. S3. The numbers of significant SNPs identified in meta-analysis within population were smaller than in single-trait GWAS. There were 17 SNPs for heifer traits and 11 SNPs for cow traits reached chromosome-wide significant level in Chinese Holsteins, and 280 SNPs were genome-wide significant with cow traits in Nordic Holsteins. The QTL detected using the meta-analysis within population are listed in Table 3. Among all QTL in meta-analysis within population, 16 were newly detected in Chinese Holsteins, while 12 were newly detected in Nordic Holsteins.

**Table 3:** The detected QTL and the most significant SNP for female fertility traits in the meta-analysis within population.

BTA	QTL boundaries					Most significant SNP^1^			
	Trait^1^	Left^2^ (bp)	Right^3^ (bp)	N^4^	Position^5^ (bp)	SNP	MAF^6^	*P*-value	Genes^7^
1	Cow_CNH	50,857,462	50,857,462	1	50,857,462	rs43235827	0.09	8.34E-06	*CBLB*
1	Cow_CNH	61,832,670	62,184,184	1	62,184,184	rs109327270	0.42	5.29E-06	*ENSBTAG00000040393*
1	Cow_NH	77,248,361	78,034,231	3	78,034,231	rs29009958	0.41	2.31E-10	*TP63*
1	Cow_NH	143,474,642	143,741,734	2	143,474,642	rs109128634	0.23	7.19E-11	*ZBTB21, PDE9A*
3	Heifer_CNH	4,668,818	6,046,690	1	6,046,690	rs43329394	0.06	1.61E-05	*NUF2*
4	Heifer_CNH	33,031,489	33,855,422	3	33,855,422	rs42429605	0.14	4.19E-06	*KIAA1324L, GRM3*
6	Heifer_CNH	18,260,174	18,260,174	1	18,260,174	rs41599462	0.03	7.83E-08	*RPL34, LEF1*
6	Cow_NH	88,592,295	88,891,318	4	88,592,295	rs110527224	0.46	4.66E-12	*ENSBTAG00000038648*
7	Cow_NH	91,483,570	91,946,384	3	91,483,570	rs110110232	0.12	5.10E-11	*ENSBTAG00000046692*
9	Heifer_CNH	27,965,979	28,149,131	3	28,018,172	rs42970704	0.39	1.85E-06	*ENSBTAG00000003656*
9	Cow_NH	53,313,101	53,405,564	3	53,313,101	rs42373432	0.31	3.81E-10	*MMS22L, KLHL32*
9	Cow_NH	89,633,289	89,847,787	1	89,762,109	rs43610539	0.39	3.80E-07	*CCDC170, ESR1*
10	Cow_NH	52,033,595	52,166,976	2	52,166,976	rs43087086	0.30	1.27E-12	*AQP9*
11	Cow_NH	76,171,277	76,744,039	2	76,171,277	rs41615369	0.22	3.24E-14	*KLHL29, ENSBTAG00000004565*
11	Cow_CNH	86,182,547	86,242,159	1	86,242,159	rs41618769	0.23	2.09E-05	*NTSR2,GREB1*
11	Cow_NH	88,712,798	88,712,798	1	88,712,798	rs110385734	0.46	2.56E-08	*ID2, ENSBTAG00000037793*
11	Cow_CNH	89,081,028	89,371,911	2	89,338,824	rs109369349	0.42	2.88E-06	*ENSBTAG00000037793, RNF144A*
12	Cow_CNH	22,219,373	22,664,255	1	22,664,255	rs29014466	0.31	1.48E-05	*FOXO1, COG6*
12	Cow_CNH	30,167,017	30,483,444	2	30,273,741	rs41571407	0.30	1.47E-05	*USPL1,HMGB1*
13	Cow_NH	33,274,673	34,124,325	12	33,918,222	rs41574065	0.13	6.60E-16	*NSUN6, EPC1*
14	Cow_NH	25,459,674	25,933,376	1	25,459,674	rs41627950	0.03	4.46E-08	*TOX*
14	Heifer_CNH	40,785,938	41,038,290	1	40,785,938	rs41629308	0.07	8.96E-06	*ENSBTAG00000047574, HNF4G*
15	Cow_NH	16,273,879	16,416,329	2	16,416,329	rs41576521	0.47	1.43E-07	*C11orf97, CWF19L2*
16	Cow_NH	43,922,935	44,459,269	2	43,922,935	rs110938771	0.27	2.05E-07	*PEX14*
16	Cow_NH	54,046,062	54,347,701	2	54,347,701	rs110082600	0.28	9.90E-08	*KAZN, PRDM2*
16	Heifer_CNH	54,368,248	54,368,248	1	54,368,248	rs109575385	0.14	2.65E-06	*KAZN, PRDM2*
17	Heifer_CNH	63,325,773	63,366,584	1	63,366,584	rs110304855	0.02	1.01E-05	*DTX1,PLBD2*
17	Cow_NH	71,381,577	71,503,614	2	71,503,614	rs109109645	0.40	5.83E-09	*SF3A1*
18	Cow_CNH	33,370,191	33,447,591	1	33,447,591	rs41581167	0.30	2.56E-05	*ENSBTAG00000037322*
18	Cow_NH	47,983,685	48,150,900	2	48,150,900	rs110543856	0.13	1.88E-09	*PRODH2, SIPA1L3*
20	Heifer_CNH	19,991,233	19,991,233	1	19,991,233	rs41638369	0.03	4.22E-06	*PDE4D*
20	Cow_NH	36,561,330	37,333,000	2	36,840,675	rs41574995	0.29	9.03E-07	*WDR70*
20	Heifer_CNH	45,940,447	45,940,447	1	45,940,447	rs41617713	0.04	3.03E-06	*CDH6, CDH9*
22	Heifer_CNH	30,245,042	30,245,042	1	30,245,042	rs517592568	0.03	1.05E-05	*FOXP1*
23	Heifer_CNH	2,247,046	2,247,046	1	2,247,046	rs109770699	0.12	1.63E-05	*KHDRBS2,PRIM2*
23	Heifer_CNH	6,068,386	6,068,386	1	6,068,386	rs110777693	0.02	3.68E-06	*TINAG, MLIP*
23	Heifer_CNH	23,098,548	23,349,487	1	23,325,266	rs110276231	0.09	2.59E-05	*TFAP2B, PKHD1*
23	Cow_CNH	50,811,981	50,923,511	1	50,811,981	rs42036487	0.09	7.72E-06	*MYLK4, ENSBTAG00000037624*
24	Cow_NH	28,877,547	29,132,144	5	29,040,145	rs42048480	0.50	3.89E-08	*CDH2*
26	Cow_NH	15,145,083	15,271,746	3	15,231,279	rs109424740	0.23	3.63E-08	*ENSBTAG00000010105*
26	Cow_NH	44,810,685	44,915,718	4	44,915,718	rs41584051	0.33	8.34E-08	*ZRANB1,CTBP2*
27	Cow_NH	1,525,307	3,721,279	4	2,748,297	rs109946086	0.28	8.26E-10	*CSMD1, ENSBTAG00000035013*
28	Cow_NH	36,010,482	36,504,079	2	36,010,482	rs109584097	0.36	1.57E-09	*FAM213A*
29	Cow_CNH	1,832,257	1,951,595	1	1,832,257	rs42494084	0.28	4.17E-06	*SLC36A4, ENSBTAG00000047614*

^1^Heifer_CNH = heifer trait in Chinese Holsteins; Cow_CNH = cow traits in Chinese Holsteins; Cow_NH = cow traits in Nordic Holsteins.

^2^The left boundary of the QTL.

^3^The right boundary of the QTL.

^4^The number of significant SNPs within the QTL.

^5^The base pair position in the BTA (*Bos taurus* autosome).

^7^Genes harbor or closest to the most significant SNP.

### Meta-analysis across populations

Manhattan plots for meta-analysis across populations are presented in Supplementary Fig. S4, while the summary of QTL for female fertility traits in the meta-analysis across populations is listed as Table 4. In total, five QTL were detected for the traits analyzed in the meta-analysis across populations: two for CRc, and each one for CRh, ICF and IFLc. The most significant QTL, segregating at 67 Mb on BTA13 for CRh harbored two significant SNPs. In general, the QTL detected in meta-analysis across populations overlapped with results from other QTL detection approaches applied in this study. For example, the QTL on BTA13 for IFLc in the meta-analysis across populations was within the genomic region 31.5~34.1 Mb, which was also significant for AISc in Chinese Holsteins, for both IFLc and ICF in Nordic Holsteins, for IFLc and ICF using the validation approach, and for cow traits in Nordic Holsteins using meta-analysis within population.

**Table 4:** The detected QTL and the most significant SNP for female fertility traits in the meta-analysis across populations.

	QTL boundaries					Most significant SNP^1^			
BTA	Trait^1^	Left^2^ (bp)	Right^3^ (bp)	N^4^	Position^5^ (bp)	SNP	MAF^6^	*P*-value	Genes^7^
1	CRc	78,034,231	78,511,271	1	78,034,231	rs29009958	0.35, 0.41	3.92E-06	*TP63*
13	IFLc	31,524,626	31,524,626	1	31,524,626	rs41604666	0.49, 0.48	3.67E-06	*RSU1*
17	CRh	67,245,920	67,941,337	2	67,245,920	rs41848120	0.48, 0.46	2.54E-06	*PIWIL3, MYO18B*
23	CRc	49,651,909	50,036,208	1	50,036,208	rs110076231	0.38, 0.45	4.79E-05	*PRPF4B, PXDC1*
28	ICF	34,021,273	34,596,949	1	34,429,786	rs110181893	0.29, 0.42	5.22E-05	*ENSBTAG00000013264, ZMIZ1*

^1^CRh = conception rate at first insemination in heifers; ICF = interval from calving to first insemination; IFLc = interval from first to last insemination in cows; CRc = conception rate at first insemination in cows.

^2^The left boundary of the QTL.

^3^The right boundary of the QTL.

^4^The number of significant SNPs within the QTL.

^5^The base pair position in the BTA (*Bos taurus* autosome).

^6^Minor allele frequency of the most significant SNP in Chinese and Nordic Holsteins.

^7^Genes harbor or closest to the most significant SNP.

### Test for multiple QTL

**Figure 1:**
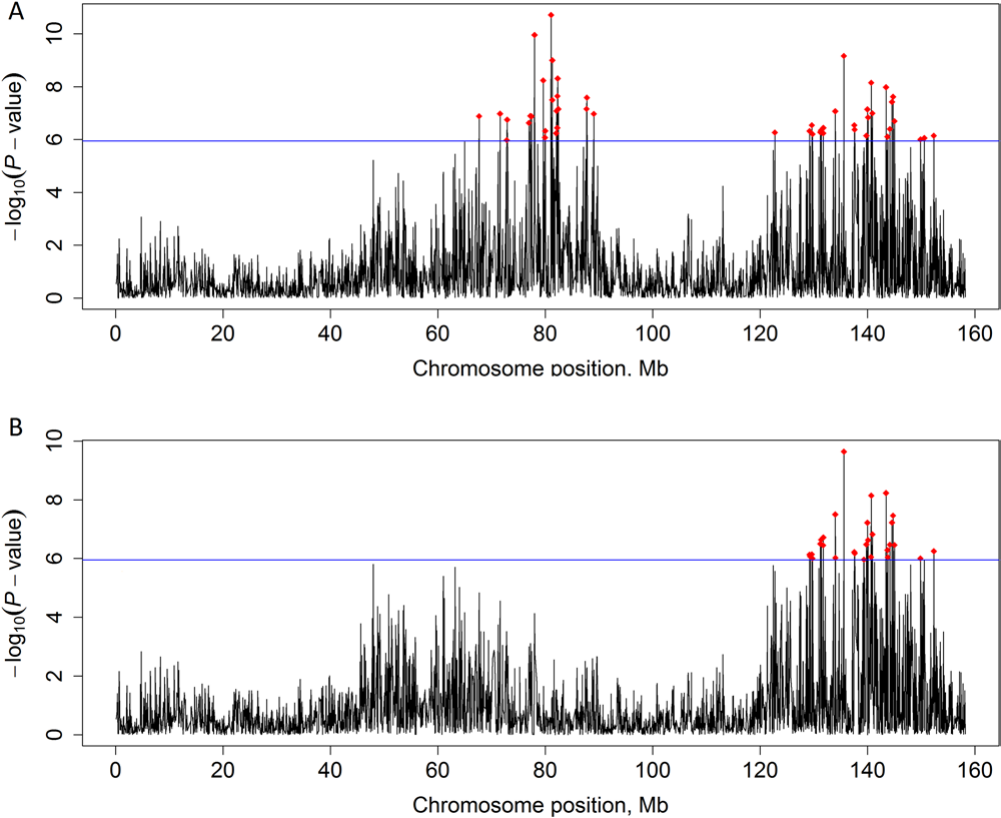
The −log (*P*-value) of SNPs on chromsosme 1 for IFLc before (**A**) and after (**B**) correcting the most significant SNP in Nordic Holsteins. The horizontal line indicates the genome-wide significance level (−log10(*P*-value) = 5.95). Genome-wide significant SNPs were hilighted using red color.

After fitting the top SNP within the chromosome into the model, the evidence for multiple QTL on the same chromosome only existed for IFLh on BTA23 in Chinese Holsteins, for IFLc on BTA1, and for ICF on BTA13 in Nordic Holsteins. For example, two large regions, including 67.7~89.1 Mb (top SNP: rs199617275, *P*-value = $1.93\times {10}^{-11}$) that contained 23 significant SNPs and 122.8~152.4 Mb (top SNP: rs41575090, *P*-value = $6.84\times {10}^{-10}$) that contained 27 significant SNPs for IFLc in Nordic Holsteins. Figure 1 shows that no other SNPs within 67.7~89.1 Mb remained significant after correcting the top SNP (rs199617275). Some SNPs in the vicinity of the SNP rs41575090, however, still reached the genome-wide significant threshold, indicating the existence of multiple QTL on BTA1 for IFLc in Nordic Holsteins.

## Discussion

The genetic architectures of female fertility traits in Chinese and Nordic Holsteins were investigated using single-trait GWAS, validation analysis and meta-analyses. The QTL discovered here not only provided valuable confirmations for previous studies^4, 7, 10, 11^, but also offered novel variants worthy of further investigation for female fertility in dairy cattle.

A lot of QTL for female fertility traits were detected using single-trait GWAS. A QTL affecting AISc in Chinese Holsteins was located at 48.5~48.9 Mb on BTA4, which was close to the QTL (near 48.9 Mb) previously detected for AISc in Nordic Holsteins^10^. The trait AISc was not available for Nordic Holsteins in this study, but the region 47.7~48.9 Mb on BTA4 was detected as a QTL for IFLc in Nordic Holsteins. Both AISc and IFLc describe capacities of cows to conceive and maintain pregnancy. The SNPs identified in present and previous studies may be in high LD with the same causative mutation. In addition, the QTL located at approximate 44.7 Mb on BTA8 contained two significant SNPs for AISc in Chinese Holsteins. QTL in similar regions were previously found in other Holstein populations. In German Holsteins, the region at 45.9 Mb on BTA8 was associated with IFL^34^. In US Holsteins, the region near 44.6 Mb harbored a QTL for daughter pregnancy rate^6^. The present and previous studies suggest that an important genetic factor for female fertility locates at 44~45 Mb on BTA8. Furthermore, the QTL on BTA11 located at 30.2~31.2 Mb for NRR56h in Chinese Holsteins was overlapped with the QTL (30.1~30.3 Mb) for age at puberty in Canadian Holsteins^35^.

In general, the power of GWAS is higher for the traits with higher heritability. In the present study, however, no significant SNP was detected for AFS which had the highest heritability among the fertility traits. The power of QTL detection, however, is not only dependent on the heritability of the trait, but also on the effect of QTL. In such a small data set as Chinese Holsteins, only genes with large effect could be detected. Therefore, a possible reason for lack of genome-wide significant SNP with AFS is that the genetic architecture underlying AFS could be a large number of genes with small effects, but no one having an effect large enough to be detected.

In Nordic Holsteins, 105 SNPs mainly located at 20~60 Mb on BTA13 showed significant associations with ICF. The importance of BTA13 on ICF was also reported in previous studies of Nordic Holsteins^7, 10, 11^. The overlapped QTL may be due to the data used in the previous studies was a subset of this study. Although various QTL detection studies have been performed previously in Nordic Holsteins, it is still necessary to perform the current GWAS for female fertility as the sample size has nearly doubled compared with the previous studies. In our study, several novel QTL were detected for female fertility due to the greater power with increased sample size, e.g. the QTL from 1,651,311 bp to 1,801,116 bp on BTA14 for CRh. This QTL harbored the gene *DGAT1*, which is generally accepted as a major gene affecting milk production traits and also with potential effects for female fertility^36^. Novel QTL detected in this study confirm that a large population size is important for QTL detection in traits with low heritabilities. Fine mapping should be performed to further narrow down these novel QTL.

The power of detecting QTL in current Chinese Holstein data was limited due to small sample size. Therefore, using a suggestive threshold in Chinese Holstein and validating associations in Nordic Holsteins is a promising approach to gain power of GWAS for Chinese Holsteins. Additional SNPs, which did not cross the stringent Bonferroni corrected significance threshold in single-trait GWAS of Chinese Holsteins, were validated in Nordic Holsteins using the validation approach. For example, the detected SNP (rs109155222) on BTA3 within gene *IL6R* was not significant neither in Chinese Holsteins (*P*-value = $4.33\times {10}^{-3}$) nor in Nordic Holsteins (*P*-value = $3.41\times {10}^{-5}$) in the respective analysis. This SNP was approximately 0.5 Mb away from the SNP segregating on 16,734,445 bp for CRh in US Holsteins^37^. The gene *IL6R* encodes a receptor of interleukin 6 complex, which is a potent pleiotropic cytokine that regulates cell growth and differentiation and plays an important role in the immune response^38^. It was reported that *IL6R* influenced the reproductive performance, embryonic development, parturition, and postnatal development in humans^39^ and mice^40^. The function of *IL6R* for female fertility in dairy cattle, however, were still less investigated than in humans and mice. In addition, four SNPs (rs110068451, rs109983109, rs110543856 and rs29027634), which were validated for more than one cow traits increased confidence for the presences of true associations with female fertility. This validation approach has been widely used in previous studies^10, 12, 34^. In the GWAS validation for production and fertility traits^12^, the SNP detected in the discovery population including 780 Holstein sires with *P*-value < 0.001 were validated in two populations: a population of 386 younger Holstein sires and a population of 364 Jersey sires. Multiple SNPs were validated for production traits in both populations, whereas no single SNP were validated for fertility in either validation population^12^. In another study for female fertility, for which Nordic Holsteins was used as the discovery population, 152 genome-wide associations were further validated in both Nordic Red and Jersey with *P*-value < 0.05^10^. In this study, some additional associated SNPs were detected using the validation approach, which support the hypothesis that validation using information of Nordic Holsteins was helpful for QTL detection for female fertility traits in Chinese Holsteins. This suggested that the validation approach could be helpful for QTL detection of low heritability traits in small population using information of other populations.

In the present study, common QTL for traits reflecting similar fertility function were detected using within population multi-trait meta-analysis. For cow traits, two QTL in Chinese Holsteins and one in Nordic Holsteins were detected on BTA11 from 86~89 Mb, which suggests that this genomic region may play similar role on fertility of cows. The gene *ID2* within this genomic region belongs to the inhibitor of DNA binding family, which is relevant to follicle maturation in the mare^41^. Besides, in Chinese Holsteins, the QTL on BTA23 with top SNP at 6,068,386 bp was significantly associated with IFLh and AISh, the association was further confirmed for heifer traits using meta-analysis within population. This QTL was close to QTL for other functional traits: abomasum displacement in Germany Holsteins^42^ and somatic cell score in Finnish Ayrshires^43^. No QTL related to female fertility traits around this QTL, however, has been reported before. Meanwhile, further studies such as the conditional analyses proposed by Bolormaa *et al*.^16^ could be performed to distinguish the significant association from meta-analysis within population was a single common QTL or multiple linked QTL.

Furthermore, some novel QTL were detected using meta-analysis within population, compared with single-trait GWAS. The QTL segregating at 89.6~89.8 Mb on BTA9 was significant for cow traits in Nordic Holsteins. The most significant SNP (rs43610539) was only 227 kb away from the gene *ESR1*, which encodes an estrogen receptor^44^. Estrogen and its receptors are essential for sexual developments and reproductive functions in cattle^44, 45^. In previous studies using meta-analysis within population, novel QTL was also detected for stature, fatness and reproduction related traits in beef cattle^16^ and for mammary gland morphology traits in German Fleckvieh cattle^46^. Novel QTL detected using meta-analysis within population demonstrated the advantage of using information from correlated traits, which also supported the hypothesis that meta-analysis within population were helpful for QTL detection for female fertility traits in Chinese Holsteins.

Some associated SNPs achieve lower *P*-values using meta-analysis across populations, when the information for the same trait from independent studies are used. For example, the *P*-value of the top SNP (rs41848120) for CRh in the meta-analysis across populations ($2.54\times {10}^{-6}$) was much lower than the *P*-value obtained from the single-trait GWAS in Chinese Holsteins ($1.28\times {10}^{-4}$) and Nordic Holsteins ($3.87\times {10}^{-5}$). Similarly, in previous studies, QTL with lower *P*-value were mapped for growth^14^ and longevity^15^ using meta-analysis across breeds (Nordic Holstein, Red dairy cattle and Jersey). Compared with results from single-trait GWAS, intervals of QTL detected by meta-analysis across population were narrower. The QTL with the same peak (rs29009958) on BTA1 for CRc from meta-analysis across populations (78,034,231~78,511,271 bp) was 0.6 Mb narrower than the one from single-trait GWAS in Nordic Holsteins (76,994,474~78,034,231 bp). The explanation could be that extensive LD persistence in dairy cattle (up to 100 kb), which might lead to the low precision of QTL region detection, was broken with the use of multiple populations^12, 47^. Lower *P*-values and narrower intervals of QTL were observed using meta-analysis across populations than using single-trait GWAS, which supported the hypothesis that meta-analysis across populations was helpful for QTL detection for female fertility traits in Chinese Holsteins. Much fewer significant SNPs, however, were observed in the meta-analysis across populations compared with single-trait GWAS, which was consistent with results of meta-analysis for feet and legs disorders^33^. This could be due to some QTL segregating only in one population. Meta-analysis is expected to be advantageous when the QTL is segregating in both population and has the effect in same direction.

In this study, the importance of the cow fertility QTL on BTA13 was not only ascertained in Nordic Holsteins, but also detected in Chinese population. The slight differences of QTL positions could be caused by the inconsistent LD pattern or inefficient QTL detection power. In the previous GWAS for Danish and Swedish Holsteins, a larger QTL segregating from 27,708,081 bp to 38,687,018 bp on BTA13 was identified for ICF and other fertility related traits, with top SNP located at 33,517,748 bp. In the previous fine mapping study for ICF, the high LD region from 33.2 Mb to 34.4 Mb was confirmed, of which the strongest signal was located at 33,903,159 bp. The genes *SLC39A12*, *CACNB2* and *ZEB1* within this genomic region were recommended as candidate genes for cow traits. The gene *CACNB2* is involved in the release of follicle-stimulating hormone from the anterior pituitary gland^48^. The gene *SLC39A12* encodes zinc transporter proteins, which could regulate the levels of free intracellular zinc in the mouse oocyte during maturation^49^. The gene *ZEB1* plays an important role in the regulation of mammalian reproduction: the lower expression of *ZEB1* leads to lower serum luteinizing hormone (LH) concentration, an impaired LH surge, and failure to ovulate^50^. Functional studies could be helpful to finally identify the causative mutations for ICF on BTA13.

The genomic region 48.3-51.9 Mb on BTA23 was detected for DO, IFLc and AISc in Chinese Holsteins, for IFLc and CRc in the validation approach, and for CRc in meta-analysis across populations. Furthermore, the region approximate 49.9 Mb on BTA23 was also a QTL for ICF, IFLc and NRR56c. Genes *PRPF4B* and *PXDC1* within this genomic region could be putative candidate genes for female fertility. The expression of *PRPF4B* in oocytes may provide supports for embryo development in cows^51^. Meanwhile, *PRPF4B* is also relevant to polycystic ovary in humans, which is a common cause of infertility^52^. The gene *PXDC1* may influence the negative energy balance in dairy cows^53^. As for the genomic region 34.0~37.6 Mb on BTA28, the QTL segregating on 34.0~34.6 Mb for ICF was close to the QTL identified for ICF in Chinese Holsteins (34.4~35.3 Mb) and Nordic Holsteins (36.0~37.6 Mb), and for cow traits in Nordic Holsteins (36.0~36.5 Mb). Genes *ZMIZ1* and *FAM213A*, which harbored the most significant SNPs, were highly recommended as significant genes for ICF. It was reported that *ZMIZ1* was relevant to diminished ovarian reserve in humans, which is one of the causes of infertility^54^. The influences of *FAM213A* for female fertility in cows have been widely discussed^48, 55^, a functional study on *FAM213A* showed that a replacement of isoleucine with valine in *FAM213A* was associated with poor fertility in cows^56^.

## Conclusions

A large numbers of QTL were discovered or confirmed for female fertility in Chinese and Nordic Holsteins in the present study. The QTL segregating at 31.4~34.1 Mb on BTA13, 48.3~51.9 Mb on BTA23 and 34.0~37.6 Mb on BTA28 shared between Chinese and Nordic Holsteins were further ascertained using the validation approach and meta-analyses. Furthermore, multiple novel variants identified in Chinese Holsteins were validated with Nordic data as well as meta-analyses. The genes *IL6R, SLC39A12, CACNB2, ZEB1*, *ZMIZ1* and *FAM213A* were concluded to be strong candidate genes for female fertility in Holsteins. Further fine mapping studies using high density SNP array and whole genome sequence variants should be performed to narrow down QTL intervals and facilitate short listing of the candidate genes.

## Electronic supplementary material

Supplemental tables and figures
