# Supplementary material for: Genome-wide Association Studies for Female Fertility Traits in Chinese and Nordic Holsteins
Source: Sci Rep. 2017 Aug 16;7:8487. doi: 10.1038/s41598-017-09170-9 (PMC5559619; doi:10.1038/s41598-017-09170-9)

Supplementary Information

Genome-wide Association Studies for Female Fertility Traits in Chinese and Nordic Holsteins

Aoxing Liu,[12] Yachun Wang,[1]* Goutam Sahana,[2] Qin Zhang,[1] Lin Liu,[3] Mogens Sandø Lund,[2] Guosheng Su[2]*

[1]Laboratory of Animal Genetics, Breeding and Reproduction, Ministry of Agriculture of China, National Engineering Laboratory of Animal Breeding, College of Animal Science and Technology, China Agricultural University, Beijing 100193, China

[2]Center for Quantitative Genetics and Genomics, Department of Molecular Biology and Genetics, Aarhus University, 8830 Tjele, Denmark

[3]Beijing Dairy Cattle Center, Beijing 100192, China

*Corresponding authors

Email addresses:

Aoxing Liu: aoxing.liu@mbg.au.dk

Yachun Wang: wangyachun@cau.edu.cn

Goutam Sahana: goutam.sahana@mbg.au.dk

Qin Zhang: qzhang@cau.edu.cn

Lin Liu: liulin@bdcc.com.cn

Mogens Sandø Lund: mogens.lund@mbg.au.dk

Guosheng Su: guosheng.su@mbg.au.dk

Supplemental tables and figures

Table S1. Estimates of variance components[1] and heritabilities (h^2) for female fertility traits in Chinese Holsteins

Trait[2]	σ_a^2	σ_{pe}^2	σ_e^2	σ_p^2	h^2 (SE)
AFS	1637.0		2424.0	4061.0	0.403(0.010)
IFLh	46.2		4277.2	4323.5	0.011(0.002)
CRh	2.126E-03		0.176	0.178	0.012(0.003)
AISh	0.019		1.371	1.390	0.014(0.003)
NRR56h	1.334E-03		0.159	0.161	0.008(0.002)
ICF	86.5	41.3	911.3	1039.1	0.083(0.006)
DO	433.8	1173.8	9034.1	10641.7	0.041(0.005)
IFLc	257.8	804.5	8438.8	9501.1	0.027(0.004)
CRc	2.314E-03	5.285E-03	0.191	0.199	0.012(0.002)
AISc	0.095	0.317	3.214	3.626	0.026(0.003)
NRR56c	1.215E-03	5.664E-03	0.210	0.217	0.006(0.001)

[1]σ_a^2 = additive genetic variance; σ_{pe}^2 = variance of permanent effect; σ_e^2= residual variance; σ_p^2= phenotypic variance.

[2]AFS = age at first insemination; IFL = interval from first to last insemination; CR = conception rate at first insemination; AIS = number of inseminations per conception; NRR56 = non-return rate at 56 d after first insemination; ICF = interval from calving to first insemination; DO = days open. For the traits expressed in both heifers and cows, a suffixes h (for heifers) or c (for cows) was attached to the trait abbreviations.

Table S2. The detected QTL (chromosome-wide significant) and the most significantly associated SNP with female fertility traits in Chinese Holsteins

	QTL boundaries				Most significant SNP[1]					
BTA	Trait[1]	Left[2] (bp)	Right[3] (bp)	N[4]	Position[5](bp)	SNP	MAF[6]	*P*-value	Effect size[7] (%)	Genes[8]
1	IFLc	125,188,443	125,237,780	2	125,237,780	rs42529951	0.14	6.28E-06	3.57	*PLOD2, C3orf58*
1	AISc	125,188,443	125,237,780	2	125,237,780	rs42529951	0.14	1.52E-05	3.05	*PLOD2, C3orf58*
3	IFLh	4,668,818	6,046,690	1	6,046,690	rs43329394	0.06	6.29E-06	1.01	*NUF2*
4	CRh	33,031,489	34,967,013	1	33,731,379	rs42426977	0.13	2.25E-05	8.21	*KIAA1324L*
4	AISc	48,460,437	48,945,677	1	48,460,437	rs109312763	0.06	5.45E-06	3.17	*PRKAR2B*
4	IFLc	52,669,435	52,690,928	2	52,690,928	rs41626104	0.02	7.64E-06	3.35	*TFEC*
4	CRc	52,624,584	52,690,928	2	52,690,928	rs41626104	0.02	7.64E-06	2.69	*TFEC*
5	ICF	15,061,346	15,105,474	1	15,105,474	rs41663008	0.10	1.75E-06	1.63	*ALX1, RASSF9*
5	CRc	111,380,409	112,494,968	1	111,909,943	rs110037094	0.39	1.51E-05	3.38	*FAM83F, TNRC6B*
6	AISh	7,363,787	7,734,092	1	7,734,092	rs109933945	0.02	1.55E-06	2.15	*SEC24D*
6	CRc	93,646,389	93,737,302	1	93,737,302	rs41571584	0.09	2.00E-05	3.28	*CCNI, CCNG2*
8	AISc	44,674,742	44,697,913	2	44,674,742	rs43550667	0.37	3.86E-06	4.23	*FOXD4L1, PGM5*
9	DO	8,425,284	8,445,313	2	8,425,284	rs41574495	0.20	2.22E-05	1.85	*ADGRB3*
11	DO	19,101,798	20,654,410	1	19,861,225	rs110633225	0.41	1.75E-05	2.90	*QPCT, RMDN2*
11	IFLc	19,101,798	20,350,454	1	19,861,225	rs110633225	0.41	1.85E-05	4.02	*QPCT, RMDN2*
11	NRR56h	30,177,813	31,173,613	1	30,177,813	rs42585748	0.02	1.05E-05	11.25	*FBXO11, FOXN2*
12	IFLh	82,450,106	84,180,733	1	82,450,106	rs110489001	0.36	4.50E-06	1.02	*ITGBL1, TPP2*
13	AISc	31,364,982	31,750,419	2	31,524,626	rs41604666	0.49	1.02E-05	3.89	*RSU1*
14	DO	19,001,879	19,629,540	1	19,531,288	rs41628482	0.36	1.12E-05	2.84	*DERL1, HAS2*
14	IFLc	19,001,879	19,715,562	1	19,531,288	rs41628482	0.36	2.22E-05	3.71	*DERL1, HAS2*
16	ICF	65,635,111	66,404,619	1	66,365,432	rs42578315	0.44	3.77E-06	1.50	*RGL1*
16	AFS	76,803,009	77,400,197	1	77,202,029	rs41826305	0.02	1.52E-05	1.09	*PLXNA2*
17	NRR56h	63,325,773	63,366,584	2	63,366,584	rs110304855	0.02	4.92E-07	12.14	*DTX1*
18	CRh	25,471,374	25,832,665	1	25,653,308	rs109742225	0.46	3.98E-05	7.88	*ADGRG5, ENSBTAG00000038141*
18	ICF	34,505,448	35,418,613	1	34,555,473	rs109481874	0.23	4.24E-05	1.24	*CMTM4*
18	DO	37,977,789	38,756,763	2	38,321,858	rs41615315	0.46	9.23E-06	3.28	*PSMD7, ZFHX3*
18	AISc	50,727,653	51,788,596	1	51,503,668	rs110850975	0.32	3.24E-05	3.60	*POU2F2, GRIK5*
18	NRR56h	60,103,953	60,135,318	1	60,135,318	rs110889414	0.25	2.91E-06	10.92	*ENSBTAG00000047053, ENSBTAG00000045985*
21	IFLc	9,064,794	9,097,550	1	9,064,794	rs42267076	0.29	1.02E-05	4.06	*ARRDC4, ENSBTAG00000046611*

21	AISc	9,064,794	9,959,262	1	9,064,794	rs42267076	0.29	2.79E-05	3.41	*ARRDC4, ENSBTAG00000046611*
21	AFS	12,408,772	12,814,108	1	12,429,328	rs108987594	0.11	3.91E-05	1.07	*MCTP2, NR2F2*
22	DO	23,390,508	24,347,814	1	23,390,508	rs110438897	0.12	1.53E-05	2.63	*CNTN4*
22	AISh	30,187,053	30,245,042	1	30,245,042	rs517592568	0.03	5.15E-07	1.95	*FOXP1*
23	IFLh	6,068,386	6,727,000	1	6,068,386	rs110777693	0.02	2.72E-05	0.78	*TINAG, MLIP*
23	AISh	6,068,386	6,517,437	1	6,068,386	rs110777693	0.02	2.10E-07	2.13	*TINAG, MLIP*
23	IFLh	23,098,548	23,445,180	1	23,325,266	rs110276231	0.09	1.02E-05	0.93	*TFAP2B, PKHD1*
23	DO	48,094,367	48,563,085	1	48,397,375	rs41610649	0.23	2.19E-05	2.65	*LY86*
23	IFLc	48,094,367	49,260,004	1	48,397,375	rs41610649	0.23	2.27E-05	3.62	*LY86*
23	AISc	48,197,978	49,260,004	1	48,397,375	rs41610649	0.23	9.83E-06	3.68	*LY86*
27	IFLc	35,084,149	36,054,950	1	35,110,230	rs109187178	0.18	4.48E-05	3.42	*IDO2, ZMAT4*
27	AISc	35,084,149	36,054,950	2	35,110,230	rs109187178	0.18	1.04E-05	3.72	*IDO2, ZMAT4*
28	AISc	9,487,766	10,919,448	1	9,827,919	rs41648518	0.22	3.60E-05	3.22	*RYR2*
28	ICF	34,429,786	35,294,673	1	35,051,834	rs41841792	0.30	1.68E-05	1.30	*ZMIZ1*

[1]AFS = age at first insemination; IFL = interval from first to last insemination; CR = conception rate at first insemination; AIS = number of inseminations per conception; NRR56 = non-return rate at 56 d after first insemination; ICF = interval from calving to first insemination; DO = days open. For the traits expressed in both heifers and cows, a suffixes h (for heifers) or c (for cows) was attached to the trait abbreviations.

[2]The left boundary of the QTL.

[3]The right boundary of the QTL.

[4]The number of significant SNPs within the QTL.

[5]The base pair position in the BTA (*Bos taurus* autosome).

[6]Minor allele frequency of the most significant SNP.

[7]The proportion of genetic variance explained by the most significant SNP.

[8] Genes harbor or closest to the most significant SNP

Table S3. The detected QTL (genome-wide significant) and the most significantly associated SNP with female fertility traits in Nordic Holsteins

	QTL boundaries				Most significant SNP[1]					
BTA	Trait[1]	Left[2] (bp)	Right[3] (bp)	N[4]	Position[5](bp)	SNP	MAF[6]	*P*-value	Effect size[7] (%)	Genes[8]
1	CRc	76,994,474	78,034,231	4	78,034,231	rs29009958	0.41	1.11E-10	0.70	*TP63*
1	IFLc	79,629,427	81,345,283	6	81,118,415	rs199617275	0.33	1.93E-11	0.73	*KNG1*
1	CRh	81,324,674	82,436,081	4	82,292,403	rs108970035	0.44	6.90E-08	0.63	*TMEM41A*
1	ICF	129,230,649	131,326,810	3	131,326,810	rs41994531	0.39	1.76E-08	0.57	*ENSBTAG00000043968*
1	IFLc	134,030,804	135,635,073	2	135,635,073	rs41575090	0.10	6.84E-10	0.69	*R3HDM1*
2	ICF	25,609,914	25,762,707	1	25,609,914	rs41580963	0.29	2.07E-07	0.55	*SMIM10L1, GAD1*
4	CRh	43,214,988	44,612,810	2	43,710,013	rs110163880	0.33	4.60E-08	0.72	*RSBN1L*
4	CRc	43,710,013	45,338,985	2	44,612,810	rs42767622	0.33	2.15E-07	0.43	*ARMC10*
4	IFLc	47,737,653	48,945,677	2	47,737,653	rs110127380	0.41	1.13E-08	0.57	*NAMPT, PIK3CG*
4	ICF	86,259,448	86,690,771	1	86,259,448	rs42764136	0.37	4.72E-07	0.51	*CPED1*
5	IFLc	109,333,059	109,843,848	1	109,843,848	rs210046780	0.42	8.38E-08	0.49	*MICAL3, PEX26*
5	CRc	117,072,865	117,072,865	1	117,072,865	rs109115893	0.32	8.08E-07	0.38	*WNT7B, PPARA*
6	CRc	66,209,350	67,193,418	2	67,193,418	rs42368174	0.48	5.51E-09	0.57	*GABRA4*
6	IFLc	82,409,949	83,256,899	2	82,409,949	rs109881581	0.34	1.27E-09	0.63	*TECRL, EPHA5*
6	ICF	88,592,295	88,891,318	4	88,592,295	rs110527224	0.46	6.34E-13	0.99	*SLC4A4, GC*
6	CRh	103,774,451	103,796,957	1	103,774,451	rs43480825	0.10	1.45E-08	0.77	*AFF1*
7	ICF	84,009,045	84,971,705	3	84,971,705	rs42450335	0.42	2.97E-08	0.55	*ENSBTAG00000013358, TMEM167A*
7	CRh	89,923,165	90,006,186	1	90,006,186	rs110208732	0.06	5.33E-07	0.48	*RASA1, TMEM161B*
7	IFLc	91,483,570	91,483,570	1	91,483,570	rs110110232	0.12	7.72E-10	0.64	*MEF2C, CETN3*
7	CRc	91,483,570	91,483,570	1	91,483,570	rs110110232	0.12	2.17E-07	0.43	*MEF2C, CETN3*
8	CRh	30,618,400	30,618,400	1	30,618,400	rs109699460	0.01	4.42E-07	0.51	*NFIB, MPDZ*
9	CRc	39,339,429	39,339,429	1	39,339,429	rs109038547	0.49	8.85E-07	0.40	*WASF1, GPR6*
9	ICF	42,792,078	42,875,324	1	42,849,970	rs109617518	0.15	4.06E-07	0.55	*SCML4*
9	IFLc	89,412,796	89,847,787	1	89,633,289	rs43612994	0.03	1.06E-06	0.40	*RMND1*
10	IFLc	52,033,595	52,166,976	2	52,166,976	rs43087086	0.30	1.04E-10	0.73	*AQP9*
10	CRc	55,719,332	56,792,715	4	55,708,101	rs41596717	0.45	8.74E-12	0.76	*UNC13C, WDR72*
10	CRh	68,474,685	69,721,690	5	69,557,467	rs109088329	0.34	2.90E-12	1.13	*OTX2, EXOC5*
11	ICF	102,974,570	103,264,921	1	103,264,921	rs110081518	0.13	2.32E-08	0.62	*ENSBTAG00000000893, ENSBTAG00000048091*

13	CRh	3,978,354	3,978,354	1	3,978,354	rs110099559	0.23	1.84E-07	0.51	*JAG1, SRY*
13	ICF	33,274,673	34,124,325	5	33,918,222	rs41574065	0.22	3.75E-17	1.14	*ARHGAP12, ZEB1*
13	IFLc	33,274,673	34,124,325	8	34,124,325	rs29020401	0.20	8.25E-11	0.71	*ZEB1*
13	CRc	38,462,778	38,462,778	1	38,462,778	rs108990317	0.27	4.87E-07	0.45	*BANF2, ENSBTAG00000003422*
13	ICF	57,548,174	57,570,093	2	57,548,174	rs41694067	0.48	1.72E-16	1.21	*PHACTR3, EDN3*
14	CRh	1,651,311	1,801,116	2	1,696,470	rs17870736	0.46	4.12E-07	0.57	*VPS28*
14	ICF	11,683,342	11,983,913	2	11,711,053	rs41583074	0.31	3.19E-07	0.51	*ASAP1*
15	ICF	8,180,827	8,808,367	1	8,180,827	rs41614029	0.33	2.54E-07	0.52	*ENSBTAG00000024648*
16	ICF	51,833,247	52,975,189	3	51,942,323	rs110485162	0.19	4.11E-09	0.64	*PRKCZ*
17	ICF	63,415,937	63,448,612	1	63,448,612	rs109593978	0.09	8.79E-07	0.45	*CFAP73, RASAL1*
17	CRh	67,245,920	67,941,337	2	67,910,146	rs108975526	0.29	8.39E-07	0.46	*MYO18B*
17	CRc	71,381,577	71,503,614	1	71,381,577	rs110123919	0.44	5.54E-09	0.59	*SF3A1*
17	IFLc	71,381,577	71,503,614	1	71,503,614	rs109109645	0.40	1.89E-10	0.71	*SF3A1*
18	ICF	47,983,685	48,150,900	1	48,150,900	rs110543856	0.13	5.84E-08	0.56	*SIPA1L3*
18	CRh	61,267,887	61,267,887	1	61,267,887	rs109118771	0.41	2.43E-07	0.52	*ENSBTAG00000000336, ENSBTAG00000046961*
18	CRc	61,267,887	61,267,887	1	61,267,887	rs109118771	0.41	1.00E-10	0.71	*ENSBTAG00000000336, ENSBTAG00000046961*
18	IFLc	61,267,887	61,267,887	1	61,267,887	rs109118771	0.41	5.37E-08	0.53	*ENSBTAG00000000336, ENSBTAG00000046961*
19	CRh	26,238,708	26,656,773	5	26,331,593	rs109928574	0.14	1.46E-07	0.72	*WSCD1, NLRP1*
21	ICF	47,273,394	47,117,318	1	47,117,318	rs110588160	0.34	5.81E-08	0.57	*MBIP, NKX2-1*
22	IFLc	46,384,288	46,810,135	2	46,533,857	rs42010971	0.45	2.62E-07	0.47	*CACNA2D3*
22	CRc	46,533,857	46,533,857	1	46,533,857	rs42010971	0.45	4.95E-07	0.41	*CACNA2D3*
22	CRh	46,533,857	46,751,219	1	46,533,857	rs42010971	0.45	9.06E-08	0.53	*CACNA2D3*
23	ICF	35,744,707	35,816,005	1	35,816,005	rs41588602	0.34	3.94E-08	0.59	*HDGFL1, SOX4*
24	CRc	7,943,787	8,007,163	1	8,007,163	rs42532273	0.38	3.50E-07	0.43	*DOK6*
24	ICF	28,877,547	29,132,144	6	29,040,145	rs42048480	0.32	2.22E-08	0.59	*CDH2*
24	IFLc	28,877,547	29,040,145	4	28,941,584	rs42761380	0.48	4.02E-08	0.54	*CDH2*
25	IFLc	757,572	930,509	1	757,572	rs109605633	0.23	1.59E-07	0.49	*LMF1*
25	ICF	28,272,468	29,532,547	2	28,272,468	rs109543600	0.31	2.27E-07	0.52	*CRCP*
28	ICF	36,010,482	37,624,697	3	36,010,482	rs109584097	0.36	4.08E-09	0.64	*FAM213A*
28	IFLc	36,010,482	37,624,697	2	36,010,482	rs109584097	0.36	2.60E-09	0.66	*FAM213A*

[1]CRh = conception rate at first insemination in heifers; ICF = interval from calving to first insemination; IFLc = interval from first to last insemination in cows; CRc = conception rate for first insemination in cows.

[2]The left boundary of the QTL. [3]The right boundary of the QTL. [4]The number of significant SNPs within the QTL. [5]The base pair position in the BTA (*Bos taurus* autosome).

[6]Minor allele frequency of the most significant SNP. [7]The proportion of genetic variance explained by the most significant SNP.

[8]Genes harbor or closest to the most significant SNP.

Figure S1. Manhattan plots for female fertility traits in Chinese Holsteins.

The horizontal line indicates the genome-wide significance level (-log10 (*P*-value) = 5.95).

AFS = age at first insemination; IFL = interval from first to last insemination; CR = conception rate at first insemination; AIS = number of inseminations per conception; NRR56 = non-return rate at 56 d after first insemination; ICF = interval from calving to first insemination; DO = days open. For the traits expressed in both heifers and cows, a suffixes h (for heifers) or c (for cows) was attached to the trait abbreviations.

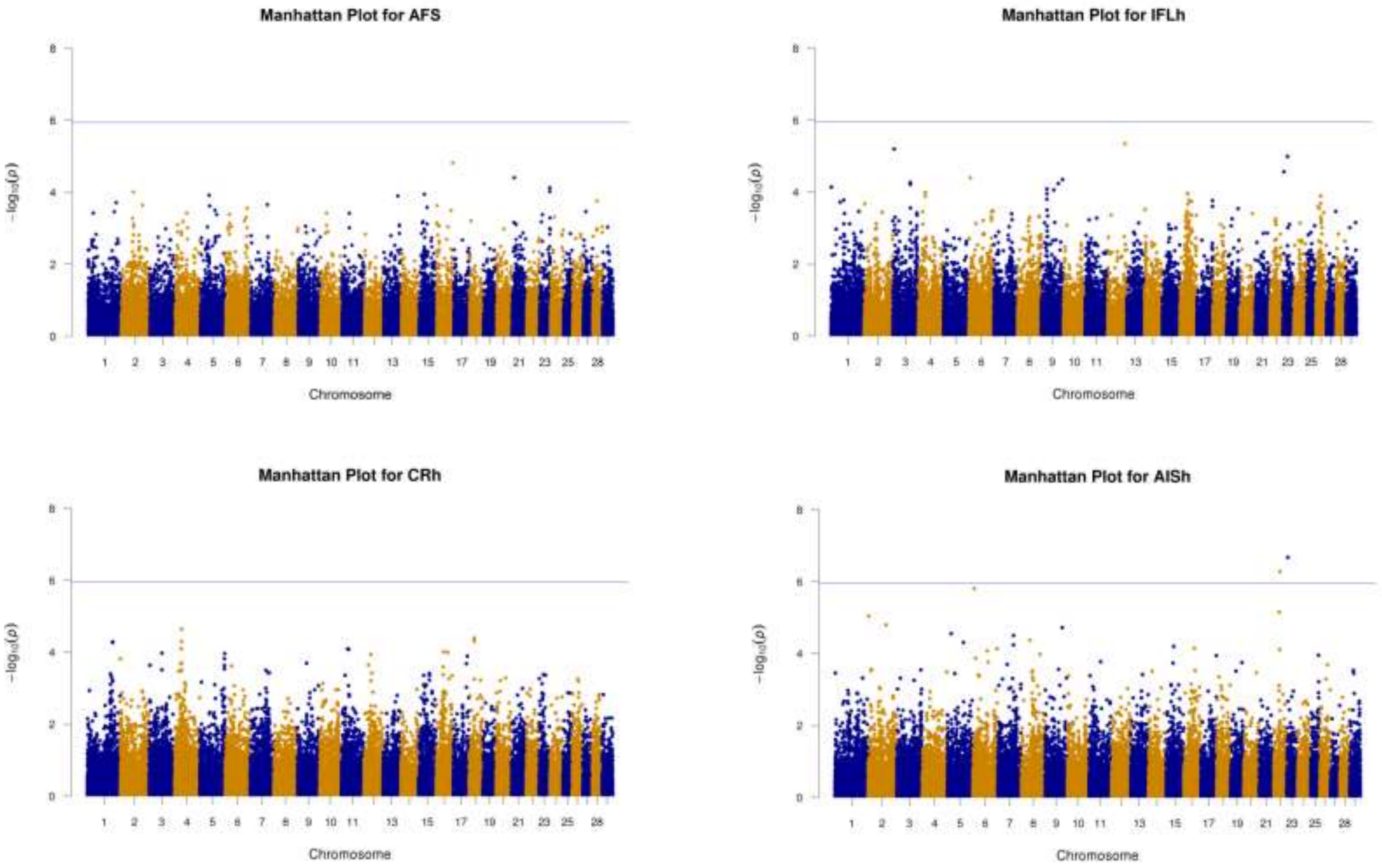

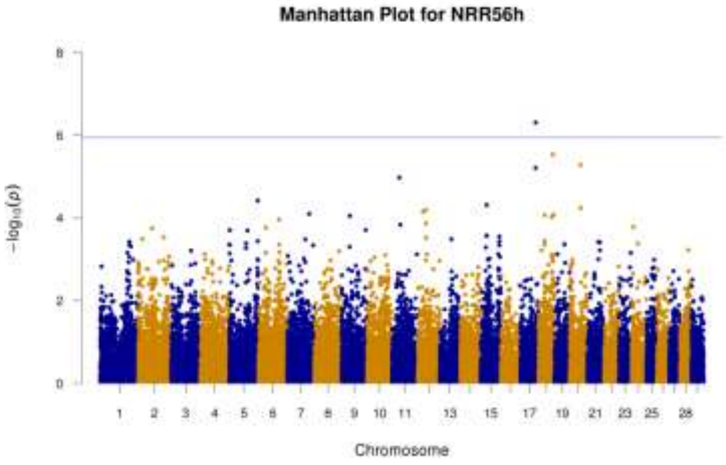
Manhattan Plot for NRR56h
$-\log_{10}(p)$
0
2
4
6
8
1 2 3 4 5 6 7 8 9 10 11 13 15 17 19 21 23 25 28
Chromosome

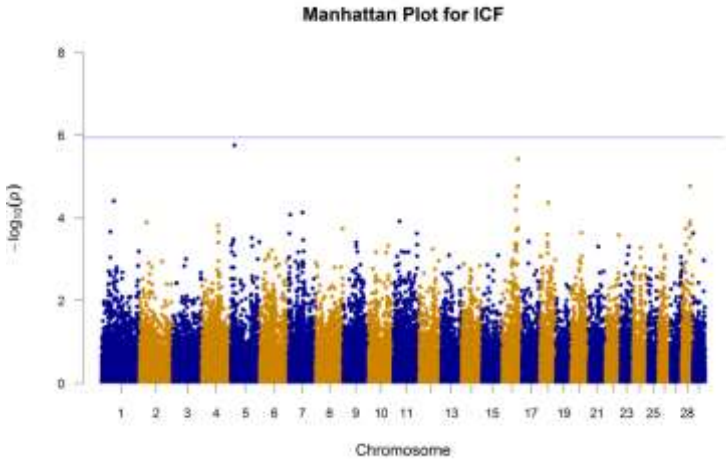
Manhattan Plot for ICF
$-\log_{10}(p)$
0
2
4
6
8
1 2 3 4 5 6 7 8 9 10 11 13 15 17 19 21 23 25 28
Chromosome

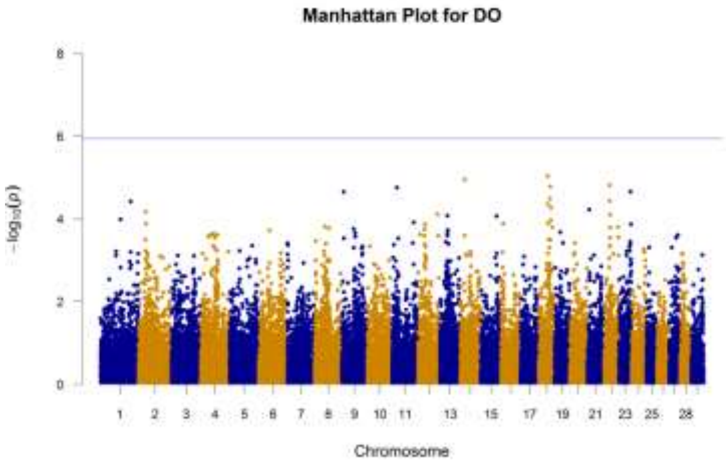
Manhattan Plot for DO
$-\log_{10}(p)$
0
2
4
6
8
1 2 3 4 5 6 7 8 9 10 11 13 15 17 19 21 23 25 28
Chromosome

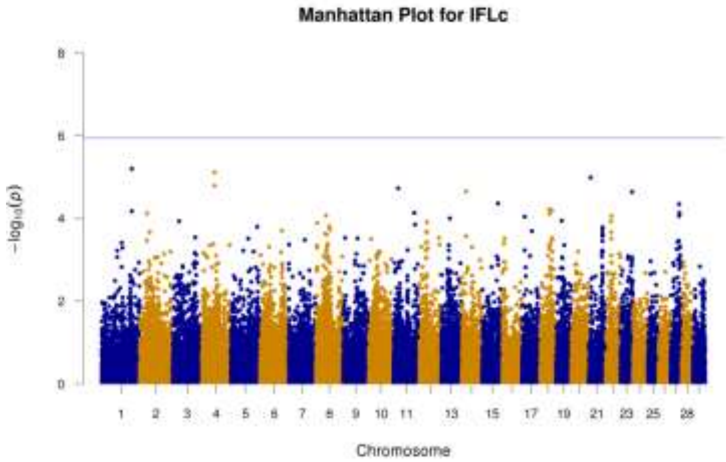
Manhattan Plot for IFLc
$-\log_{10}(p)$
0
2
4
6
8
1 2 3 4 5 6 7 8 9 10 11 13 15 17 19 21 23 25 28
Chromosome

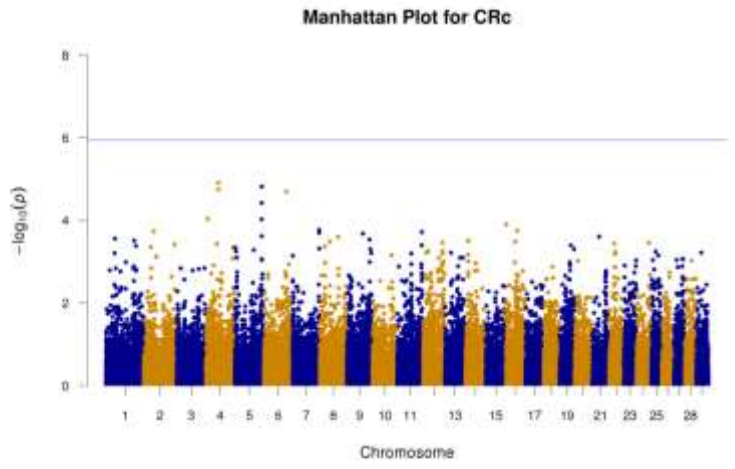
Manhattan Plot for CRc
−log10(p)
Chromosome

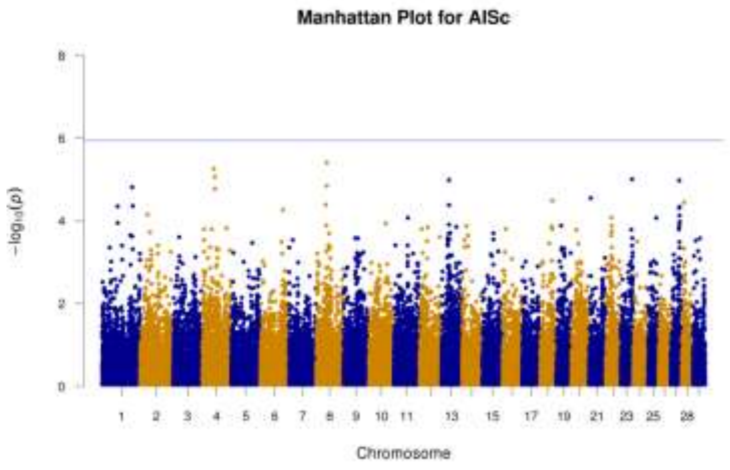
Manhattan Plot for AISc
−log10(p)
Chromosome

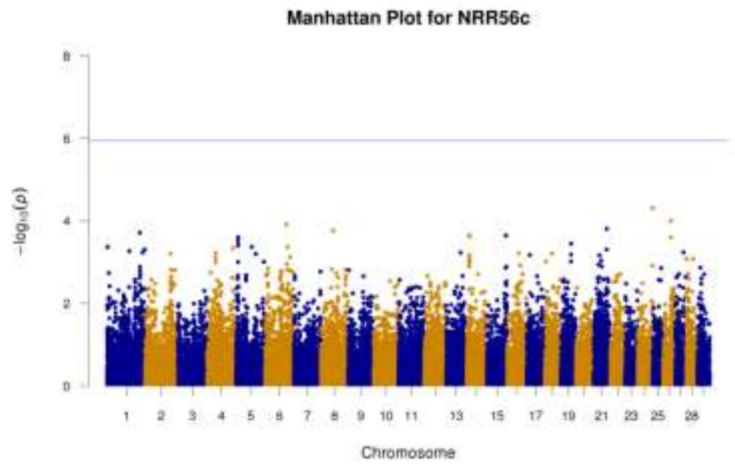
Manhattan Plot for NRR56c
−log10(p)
Chromosome

Figure S2. Manhattan plots for female fertility traits in Nordic Holsteins.

The horizontal line indicates the genome-wide significance level (-log10 (P-value) = 5.95).

CRh = conception rate at first insemination in heifers; ICF = interval from calving to first insemination; IFLc = interval from first to last insemination in cows; CRc = conception rate for first insemination in cows.

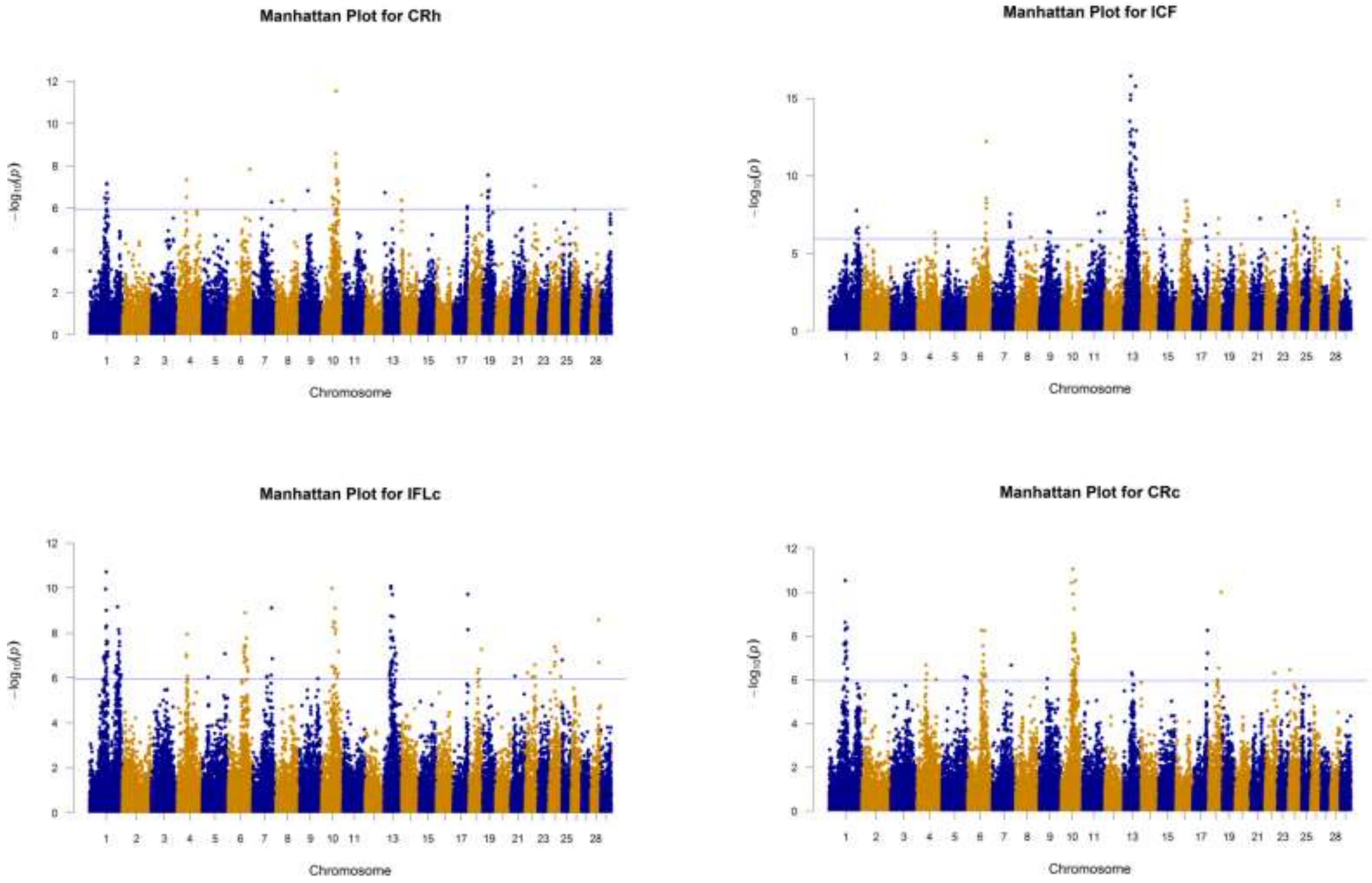

Figure S3. Manhattan plots for female fertility traits using multi-trait meta-analysis within population.
The horizontal line indicates the genome-wide significance level (-log10 (P-value) = 5.95).
Heifer_CHN = heifer trait in Chinese Holsteins; Cow_CHN = cow traits in Chinese Holsteins; Cow_NH = cow traits in Nordic Holsteins.

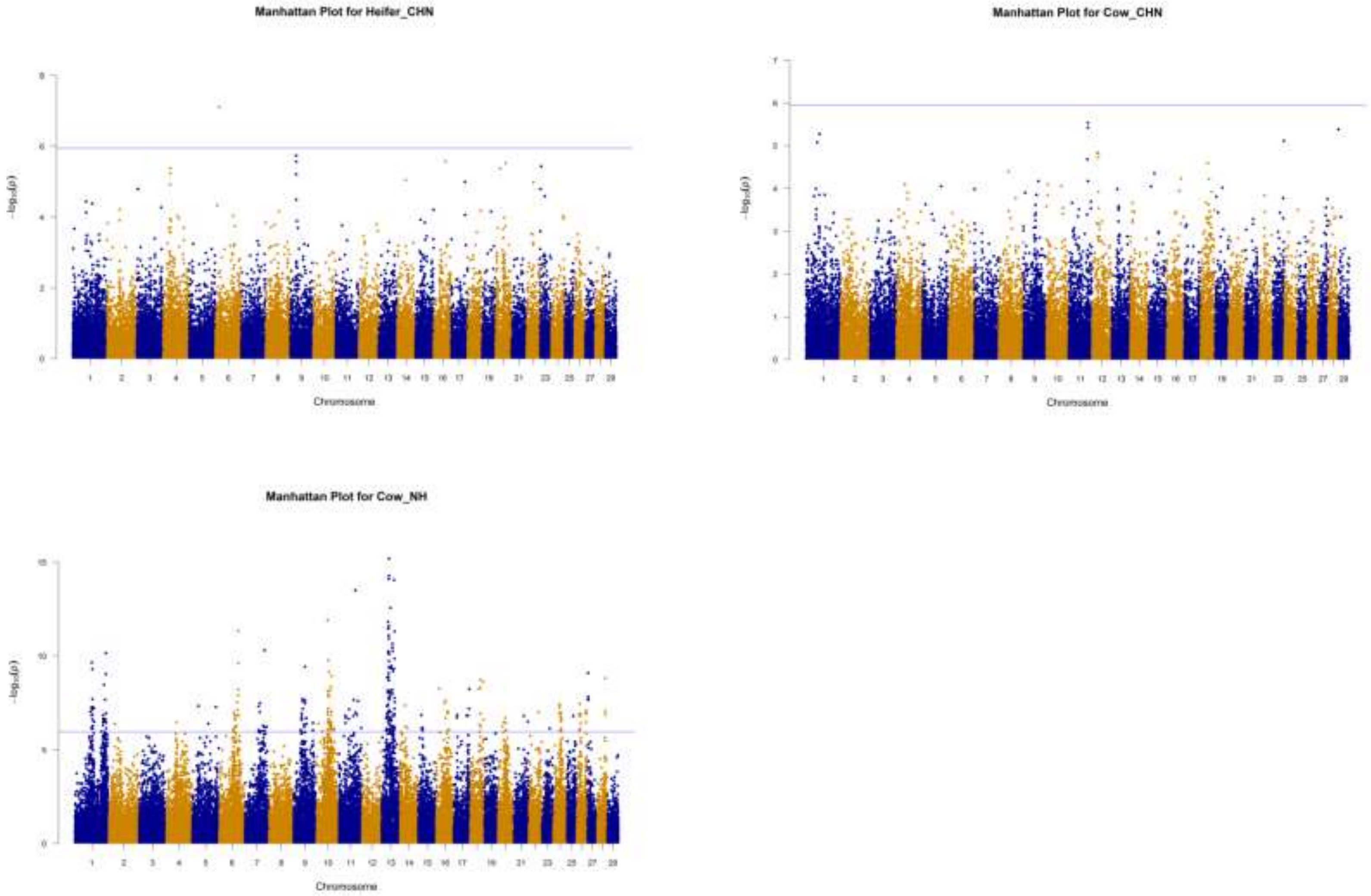

Figure S4. Manhattan plots for female fertility traits using meta-analysis across populations.

The horizontal line indicates the genome-wide significance level (-log10 (P-value) = 5.95).

CRh = conception rate at first insemination in heifers; ICF = interval from calving to first insemination; IFLc = interval from first to last insemination in cows; CRc = conception rate at first insemination in cows.

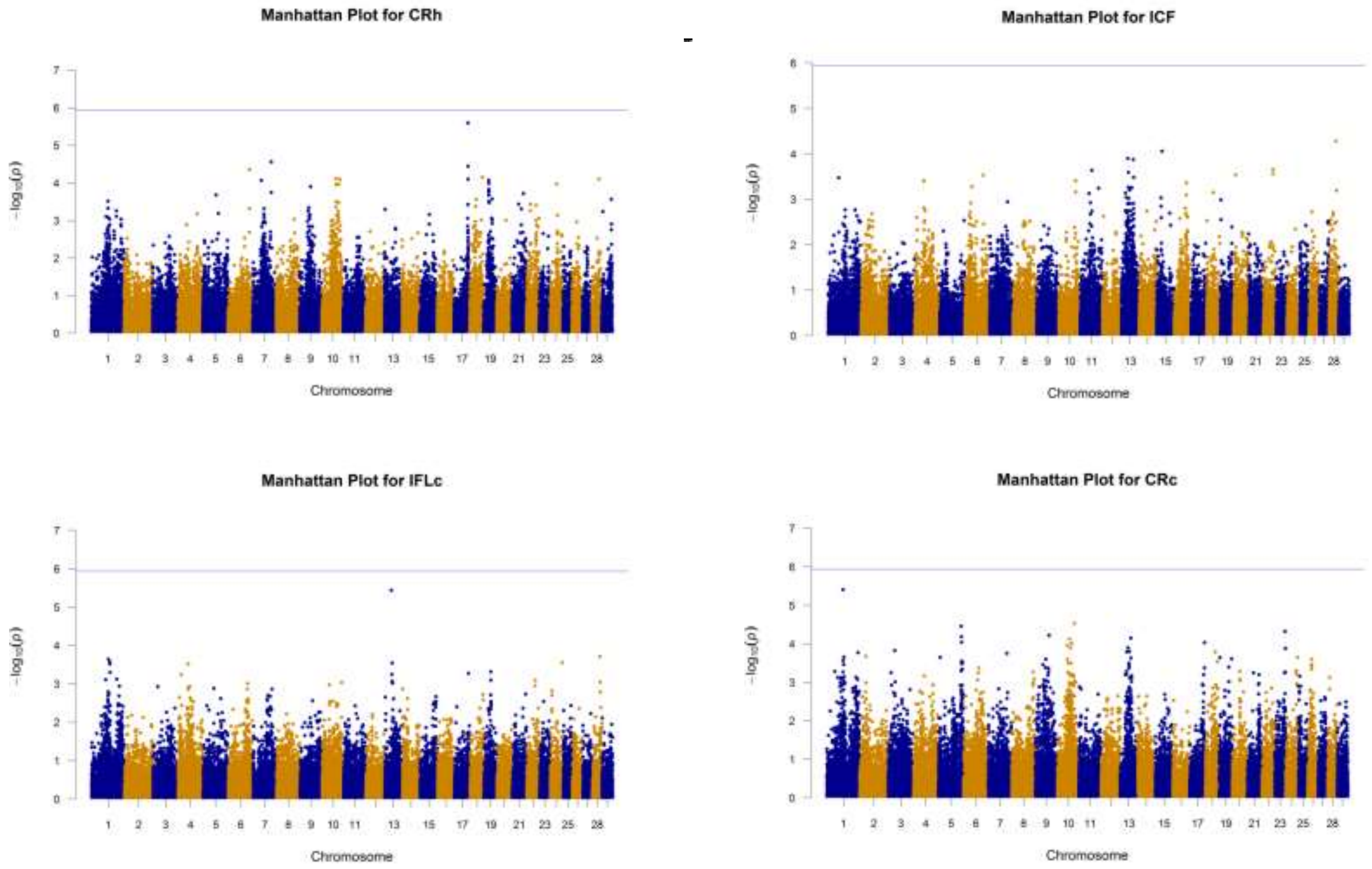

Supplement: Supplementary file 1 — Supplemental tables and figures [file 41598_2017_9170_MOESM1_ESM.pdf]
